# Systemic RAGE ligands are upregulated in tuberculosis individuals with diabetes co-morbidity and modulated by anti-tuberculosis treatment and metformin therapy

**DOI:** 10.1186/s12879-019-4648-1

**Published:** 2019-12-09

**Authors:** Nathella Pavan Kumar, Kadar Moideen, Arul Nancy, Vijay Viswanathan, Basavaradhya S. Shruthi, Shanmugam Sivakumar, Syed Hissar, Hardy Kornfeld, Subash Babu

**Affiliations:** 1 National Institutes of Health—NIRT— International Center for Excellence in Research, No. 1 Mayor Sathyamoothy Road, Chetpet, Chennai, India; 2 Prof. M. Viswanathan Diabetes Research Center, Chennai, India; 3 National Institute for Research in Tuberculosis, Chennai, India; 4 University of Massachusetts Medical School, Worcester, MA USA; 5 LPD, NIAID, NIH, Bethesda, MD USA

**Keywords:** *Mycobacterium tuberculosis*, Diabetes mellitus, RAGE ligands

## Abstract

**Background:**

Ligands of the receptor for advanced glycation end products (RAGE) are key signalling molecules in the innate immune system but their role in tuberculosis-diabetes comorbidity (TB-DM) has not been investigated.

**Methods:**

We examined the systemic levels of soluble RAGE (sRAGE), advanced glycation end products (AGE), S100A12 and high mobility group box 1 (HMGB1) in participants with either TB-DM, TB, DM or healthy controls (HC).

**Results:**

Systemic levels of AGE, sRAGE and S100A12 were significantly elevated in TB-DM and DM in comparison to TB and HC. During follow up, AGE, sRAGE and S100A12 remained significantly elevated in TB-DM compared to TB at 2nd month and 6th month of anti-TB treatment (ATT). RAGE ligands were increased in TB-DM individuals with bilateral and cavitary disease. sRAGE and S100A12 correlated with glycated hemoglobin levels. Within the TB-DM group, those with known diabetes (KDM) revealed significantly increased levels of AGE and sRAGE compared to newly diagnosed DM (NDM). KDM participants on metformin treatment exhibited significantly diminished levels of AGE and sRAGE in comparison to those on non-metformin regimens.

**Conclusions:**

Our data demonstrate that RAGE ligand levels reflect disease severity and extent in TB-DM, distinguish KDM from NDM and are modulated by metformin therapy.

## Background

The co-prevalence of tuberculosis (TB) and diabetes mellitus (DM) has grown into a major barrier to TB elimination. DM and TB are foremost killers of mankind around the globe [1]. Since DM increases the risk of progress of latent TB infection (LTBI) to active tuberculosis threefold [2, 3], the coexistence of the two diseases jeopardizes global health and justifies routine bidirectional screening [4]. DM affects the immune system by worsening both innate and adaptive immune functions, which in turn leads to increased risk of poor TB outcomes plus increased transmission, poorer clinical presentation, treatment failure and death [5–10].

RAGE is a multiligand receptor of the immunoglobulin superfamily involved in inflammation, DM and its associated complications [11, 12]. Of the defined pathways implicated in the pathogenesis of diabetic complications, RAGE receptor signalling has been the most widely studied in leukocytes, immune function, and response to infection. The RAGE receptor signals through binding of not only AGEs, as the name implies, but also the A8, A9 and A12 members of S100 protein family, the cellular stress signalling protein, high mobility group box 1 (HMGB-1), and β-amyloid sheets [13]. All of these ligands are either direct products of high glucose or are induced by the cellular stress of hyperglycemia [14]. The formation of RAGE ligands might be relevant to TB-DM comorbidity because RAGE ligands are known to accumulate to a greater level with DM and also in the presence of chronic inflammation, such as that which occurs in TB [12]. It is possible that RAGE ligands may accumulate at a faster rate in people with TB-DM, given the convergence of hyperglycemia and chronic inflammation. RAGE ligand upregulation may alter immune cell function and lead to a prolonged proinflammatory response through RAGE-mediated activation of nuclear factor kappa-light-chain-enhancer of activated B cells (NF-kB) [15, 16].

In our current data, we elucidated the systemic levels of RAGE ligands at baseline and at two time-points following initiation of anti-TB treatment (ATT): 2 months, which marks the end of the intensive phase, and 6 months, when treatment is completed. Our data demonstrate that DM differentially modulates the circulating RAGE ligands in participants with TB before, during and after completion of anti-TB treatment. Our current findings also reveal that systemic RAGE ligands levels indicate baseline disease severity and extent in TB-DM, discriminate KDM from NDM and are altered by ATT and metformin therapy.

## Methods

### Study population

We recruited and collected the plasma samples from a group of active pulmonary TB individuals with diabetes mellitus (TB-DM) (*n* = 44), individuals with active pulmonary TB (TB) alone (*n* = 44), individuals with diabetes mellitus (DM) alone (n = 44) and healthy control individuals with no TB or diabetes (HC) ) (n=30) recruited in Chennai, India (Table 1). This was the same set of individuals previously used for studying the association of monocyte activation markers with TB-DM and we used the same methodology previously described by Kumar NP et al., [17]. Pulmonary TB cases were microbiologically confirmed based on smear and culture positivity for *Mycobacterium tuberculosis* (*M.tb)*. For defining cavitary disease and lung lesions, chest X-rays were used. The breakup is as follows: cavitary disease (TB-DM, *n* = 13 and TB, *n* = 10) and non-cavitary disease (TB-DM, *n* = 31 and TB, *n* = 34) as well as unilateral (TB-DM, *n* = 24 and TB, *n* = 25) versus bilateral (TB-DM, *n* = 20 and TB, *n* = 19) lung involvement. Bacterial burdens were estimated using the AFB smear grades and classified as 1+ (TB-DM, *n* = 14 and TB, n = 19), 2+ (TB-DM, n = 19 and TB, n = 14) and 3+ (TB-DM, *n* = 11 and TB, n = 11). All the active TB cases had no record of prior TB disease or ATT during the time of enrolment. Oral glucose tolerance test and/or glycated hemoglobin (HbA1c) levels (for known diabetics) were used for diagnosing the glycemic status (DM or normoglycemia) according to the WHO criteria. Among the 44 TB-DM individuals, 22 were known diabetics (KDM) and 22 were newly diagnosed diabetics (NDM). Patients with prior history of diabetes were confirmed by HbA1c testing and grouped as known diabetic (KDM). Those with no prior history of diabetes were assessed by fasting plasma glucose (FPG) test and oral glucose tolerance test (OGTT) (75-g glucose challenge). Glycemic status based on plasma glucose 2 h post-challenge was determined according to World Health Organization (WHO) criteria: DM (> 200 mg/dL), impaired glucose tolerance (140 to 199 mg/dL), normoglycemia (< 140 mg/dL). These individuals were grouped as NDM and the diagnosis was made at the time of TB diagnosis. Among the KDM individuals, patients were classified as those who were taking metformin containing anti-diabetic medication (*n* = 11) and patients who were on insulin or glimepiride (*n* = 11) (Table 2). All the recruited DM and HC individuals were negative for Quantiferon TB gold assay with no clinical symptoms of TB and normal chest X-rays. To all the enrolled TB-DM and TB individuals, standard ATT using the directly observed treatment, short course (DOTS) strategy was administered. At the end 2 and 6 months of ATT, fresh plasma samples were collected from TB-DM and TB individuals. All the enrolled TB-DM and TB individuals were culture negative at the end of ATT.

**Table 1:** Demographic and clinical variables of the study groups and biochemical parameters in TB-DM, TB, DM and HC

Study Demographics	TB-DM		TB	DM	HC
	KDM	NDM			
Number of subjects recruited	22	22	44	44	30
Gender (Male / Female)	16/6	18/4	27/17	30/14	15/15
Median Age (Range)	52 (25–70)	42 (29–70)	39 (24–67)	44 (33–68)	34 (23–55)
BMI kg/m^2^	21.7 (13.2–28.6)	17.2 (13.2–32.6)	16.5 (13.2–30.1)	20.1 (14.2–32.1)	20.1 (18.2–25.3)
Smear Grade: 0/1+/2+/3+	0/4/10/8	0/10/9/3	0/19/14/11	NA	NA
Cavitary Disease (Y/N)	9/13	4/18	10/34	NA	NA
Lung Lesions (Unilateral/Bilateral)	13/9	11/11	25/19	NA	NA
Fasting Blood Glucose, mg/dL	166 (120–417)	139 (111–375)	93 (73–103)	144 (95–405)	75 (70–109)
Post-prandial Glucose, mg/dL	350 (217–550)	311 (202–505)	110 (68–137)	341 (210–543)	98 (72–139)
Glycated hemoglobin level, %	12.3 (8–15.6)	10 (7.3–13.9)	5.6 (5.0–5.8)	10 (6.8–12.6)	5.5 (5.0–5.9)

The values represent the geometric mean (and the 95% confidence intervals) except for age where the median (and the range) are depicted

### Elisa

Circulating levels of AGE (carboxymethyl lysine) were measured using the Cell Biolabs INC kit. Soluble RAGE (sRAGE) was measured using the Quantikine ELISA kit (R&D Systems), S100A12 using the MBL international corporation kit and HMGB-1 using the Mybiosource kit. The lowest detection limits were as follows: AGE, 0.39 μg/mL; sRAGE, 78.12 pg/mL; S100A12, 20 pg/mL and HMGB-1, 19.5 pg/mL.

### Statistical analysis

For measuring the central tendency, Geometric means (GM) were used. Kruskal-Wallis test with Dunn’s correction for multiple comparisons were used for analysing the statistically significant differences between the four groups. The Mann-Whitney test was used to compare RAGE ligand concentrations between TB patients with and without DM, unilateral or bilateral lung lesions and cavitary or non-cavitary disease. Linear trend post-test was used to compare RAGE ligand concentrations with smear grades (reflecting bacterial burdens) and Spearman rank correlation was used to compare RAGE ligand concentrations with HbA1c levels. Analyses were performed using GraphPad PRISM Version 7.

## Results

### Study population characteristics

The baseline characteristics including demographic and biochemical features of the study population are shown in Table 1. No significant differences were observed in age, sex, smear or culture grades at baseline between the TB-DM and TB groups (Table 1).

**Table 2:** Demographic and clinical variables of the study groups and biochemical parameters in KDM individuals who are on metformin and non-metformin treatment

Study Demographics	TB-DM	
	KDM	
	Metformin	Non-metformin
No. of subjects recruited	11	11
Gender (Male/Female)	8/3	8/3
Median Age (Range)	50 (25–70)	48 (30–65)
BMI kg/m^2^	20.85 (15.5–28.6)	20.97 (13.8–26.1)
Diabetic Medications	Metformin	Insulin+Glimepiride
Smear Grade: 0/1+/2+/3+	0/3/4/4	0/1/6/4
Cavitary Disease (Y/N)	4/7	5/6
Lung Lesions (Unilateral/Bilateral)	7/4	6/5
Glycated hemoglobin level, %	11 (7.7–15.6)	10.2 (7.9–14.3)

The values represent the geometric mean (and the 95% confidence intervals) except for age where the median (and the range) are depicted

### Elevated levels of circulating RAGE ligands in TB-DM

**Fig. 1:**
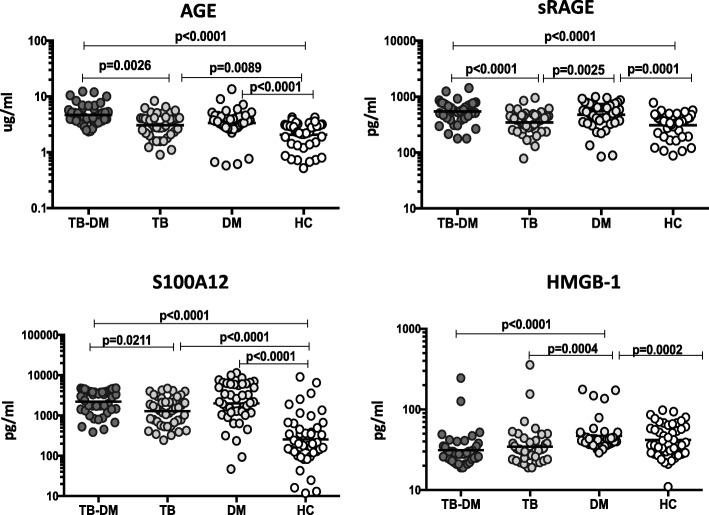
Enhanced circulating levels of RAGE ligands in TB-DM and DM participants. The plasma levels of AGE, sRAGE, S100A12 and HMGB-1 were measured in TB-DM (*n* = 44), TB (*n* = 44), DM (*n* = 44) and HC (*n* = 30) individuals at baseline. The data are illustrated as scatter plots with each circle representing a single participant. *P* values were calculated using the Kruskal-Wallis test with Dunn’s post-hoc for multiple comparisons

To elucidate the effect of TB and DM on systemic RAGE ligand expression, we estimated the plasma levels of AGE, sRAGE, S100A12 and HMGB-1 in TB-DM, TB, DM and HC individuals (Fig. 1). Plasma levels of AGE (Geo Mean 4.65 pg/ml in TB-DM vs 2.93 pg/ml in TB, 3.25 pg/ml in DM and 2.04 pg/ml in HC), sRAGE (Geo Mean 541.2 pg/ml in TB-DM vs 345.6 pg/ml in TB, 475.1 pg/ml in DM and 307.7 pg/ml in HC) and S100A12 (Geo Mean 2222 pg/ml in TB-DM vs 1286 pg/ml in TB, 2018 pg/ml in DM and 254 pg/ml in HC) were significantly increased in TB-DM and DM in comparison with TB and HC study participants. In contrast, the circulating levels of HMGB-1 (Geo Mean 31.1 pg/ml in TB-DM vs 34.5 pg/ml in TB, 46.8 pg/ml in DM and 41.7 pg/ml in HC) was significantly higher in DM in comparison with TB-DM, TB and HC. Thus, DM with or without coexisting TB disease was associated with significantly increased plasma levels of RAGE ligands and sRAGE, while only DM was associated with elevated HMGB-1.

### Elevated circulating levels of RAGE ligands in TB-DM compared to TB during ATT

**Fig. 2:**
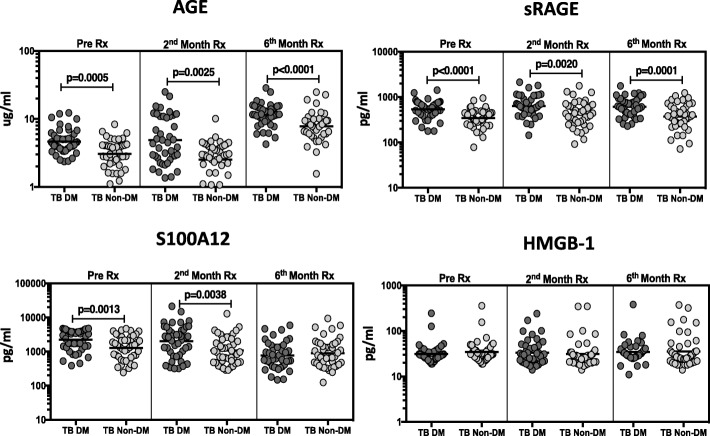
TB-DM is associated with increased frequencies of RAGE ligands at pre-treatment and following ATT. The plasma levels of RAGE ligands in TB-DM (*n* = 44) and TB (*n* = 44) participants at pre-treatment and at two and six months following ATT. The data are illustrated as scatter plots with each circle representing a single participant. *P* values were calculated using the Mann-Whitney test

To elucidate whether the RAGE ligands or sRAGE were changed by anti-TB treatment, we estimated the circulating plasma levels of AGE, sRAGE, S100A12 and HMGB-1 in TB-DM and TB at baseline (pre-treatment), during treatment (2nd month) and at the completion of ATT (6th month). As shown in Fig. 2, AGE (Geo Mean of 4.88 pg/ml in TB-DM vs 2.52 pg/ml in TB); sRAGE (Geo Mean 635.2 pg/ml in TB-DM vs 425.6 pg/ml in TB) and S100A12 (Geo Mean 2018 pg/ml in TB-DM vs 1073 pg/ml in TB) levels remained significantly increased in TB-DM compared to TB at 2 months treatment. The differentially elevated levels of AGE (Geo Mean 11.5 pg/ml in TB-DM vs 7.8 pg/ml in TB) and sRAGE (Geo Mean 614.6 pg/ml in TB-DM vs 369.6 pg/ml in TB) persisted through the completion of ATT, whereas the difference in S100A12 levels (Geo Mean 773.1 pg/ml in TB-DM vs 897.7 pg/ml in TB) was extinguished by month 6. This result suggests that elevation of AGE and sRAGE are intrinsic to the diabetic state, whereas S100A12 may be at least partially regulated by TB disease activity.

### Circulating RAGE ligands as markers of disease severity in TB-DM

**Fig. 3:**
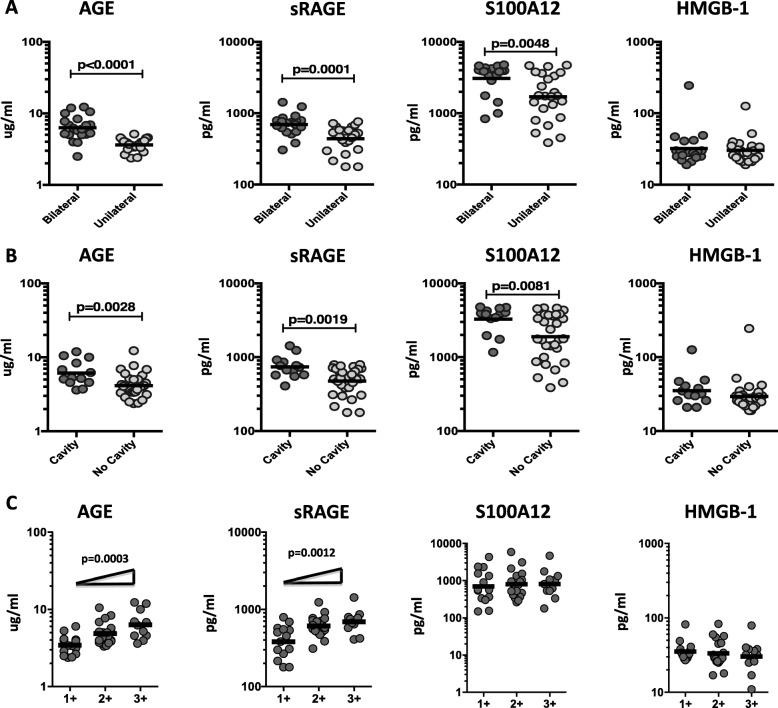
Increased circulating levels of AGE and sRAGE in bilateral and cavitary disease and also marker of bacterial burden in TB-DM participants. **a** The plasma levels of AGE, sRAGE, S100A12 and HMGB-1 were measured in TB-DM individuals with bilateral versus unilateral disease. **b** The plasma levels of AGE, sRAGE, S100A12 and HMGB-1 were measured in TB-DM individuals with cavitary versus non-cavitary disease. **c** The relationship between the plasma levels of AGE, sRAGE, S100A12 and HMGB-1 and smear grades as measured by sputum smears was examined in TB-DM individuals. The data are illustrated as scatter plots with each circle representing a single participant. P values were calculated using the Mann-Whitney test with Holm’s correction for multiple comparisons. For bacterial burden correlation, P values were calculated using the Linear trend post-test

To elucidate the relationship between the circulating plasma levels of RAGE ligands and disease severity in TB-DM, based on chest X-ray we compared plasma levels of AGE, sRAGE, S100A12 and HMGB-1 in TB-DM and TB study participants with unilateral vs bilateral disease and cavitary vs non-cavitary disease. As shown in Fig. 3a, the systemic plasma levels of AGE (Geo Mean 6.28 pg/ml in bilateral vs. 3.62 pg/ml in unilateral disease), sRAGE (Geo Mean 693.4 pg/ml in bilateral vs. 440.7 pg/ml in unilateral disease) and S100A12 (Geo Mean 3079 pg/ml in bilateral vs. 1693 pg/ml in unilateral disease) were significantly increased in TB-DM individuals with bilateral disease in comparison with unilateral disease. In contrast, no significant differences were seen between bilateral vs unilateral disease in TB individuals without diabetes (Additional file 1: Table S1). As shown in Fig. 3b, systemic plasma levels of AGE (Geo Mean 6.13 pg/ml in cavitary vs. 4.14 pg/ml in non-cavitary disease), sRAGE (Geo Mean 737.9 pg/ml in cavitary vs. 475.6 pg/ml in non-cavitary disease) and S100A12 (Geo Mean 3264 pg/ml in cavitary vs. 1891 pg/ml in non-cavitary disease) were significantly increased in TB-DM individuals with cavitary disease in comparison with those without. In contrast, no significant differences were seen between cavitary vs non-cavitary disease in TB individuals without diabetes (Additional file 2: Table S2). To determine the association of the systemic levels of RAGE ligands and bacterial burdens, we performed a correlation of the circulating levels of AGE, sRAGE, S100A12 and HMGB-1 in TB-DM and TB individuals with smear grades. As shown in Fig. 3c, both AGE and sRAGE displayed a significant positive relationship with smear grades in TB-DM and TB individuals, indicating a positive association of these factors with bacterial burdens. Thus, disease severity assessed radiographically and by estimated bacterial burden in TB-DM was associated with elevated systemic levels of RAGE ligands.

### Circulating RAGE ligands reveal a positive relationship with HbA1c in TB-DM, are increased in KDM individuals and decreased by metformin treatment

**Fig. 4:**
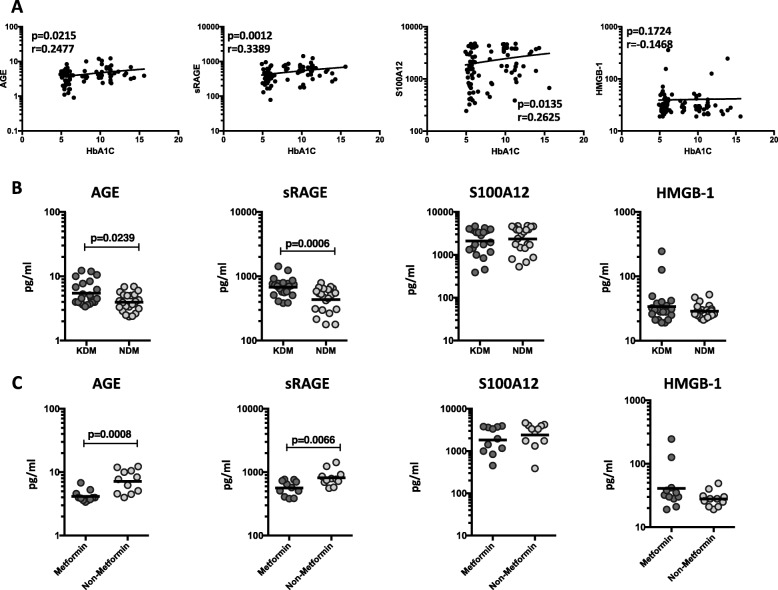
Significant correlation between circulating levels of RAGE ligands and glycemic parameters and elevated circulating levels of RAGE ligands in KDM individuals. **a** The relationship between the plasma levels of AGE, sRAGE, S100A12 and HMGB-1 and HbA1c levels was examined in all TB participants with and without DM at baseline. **b** The plasma levels of AGE, sRAGE, S100A12 and HMGB-1 were measured in TB-DM individuals with known diabetes (KDM) versus newly diagnosed diabetes (NDM) (**c**) The plasma levels of AGE, sRAGE, S100A12 and HMGB-1 were measured in KDM individuals on metformin treatment versus no metformin treatment. The data are illustrated as scatter plots with each circle representing a single participant. For HbA1c correlations, P values were calculated using the Spearman Rank Correlation. For KDM, P values were calculated using the Mann-Whitney test with Holm’s correction for multiple comparisons

To elucidate the relationship between circulating plasma levels of RAGE ligands and glycemic control in TB-DM, we determined the association between the systemic levels of baseline HbA1c with AGE, sRAGE, S100A12 and HMGB-1 in all TB participants with and without DM (Fig. 4a). As shown, the circulating levels of AGE, sRAGE and S100A12 showed a significant weak positive relationship with HbA1c, implying an association of these factors with poor glycemic control. Interestingly, this significant correlation is abolished upon completion of ATT, suggesting that TB per se has an effect in modulating either RAGE ligands or HbA1c levels or both. To estimate whether RAGE ligands can distinguish between KDM (*n* = 22) and NDM (*n* = 22) in TB-DM participants, we first determined the HbA1c levels in KDM and NDM and observed that HbA1c % was significantly increased in KDM in comparison with NDM (Geometric Mean 11.4% in KDM vs 8.9% in NDM, *p* = 0.0028). Second, we estimated the baseline levels of AGE, sRAGE, S100A12 and HMGB-1 in KDM and NDM individuals. As shown in Fig. 4b, circulating plasma levels of AGE (Geo Mean 5.46 pg/ml in KDM vs. 3.96 pg/ml in NDM) and sRAGE (Geo Mean 675.3 pg/ml in KDM vs. 434.3 pg/ml in NDM) were significantly increased in KDM in comparison with NDM participants. Thus, KDM is linked with increased circulating plasma levels of RAGE ligands at baseline. Since previously published studies reported that anti-diabetic drug metformin is correlated with protection against mortality in TB-DM, [18] we estimated the systemic levels of AGE, sRAGE, S100A12 and HMGB-1 in KDM individuals on metformin treatment (*n* = 11) in comparison with those on non-metformin regimens (*n* = 11). Among the KDM study participants on metformin regimen compared to KDM individuals not on metformin regimen, we found no significant differences in HbA1c levels. As shown in Fig. 4c, circulating plasma levels of AGE (Geo Mean 4.16 pg/ml in Metformin vs. 7.16 pg/ml in Non-Metformin) and sRAGE (Geo Mean 560.8 pg/ml in Metformin vs. 813.1 pg/ml in Non-Metformin) were significantly decreased in KDM individuals on metformin regimen in comparison with KDM individuals not on metformin. Thus, metformin therapy in KDM individuals is linked with decreased circulating plasma levels of AGE and sRAGE, but no change in the RAGE ligands, S100A12 or HMGB-1.

### Systemic RAGE ligands exhibit relationship with pro-inflammatory cytokines

We have previously measured the systemic plasma levels of pro-inflammatory cytokines (IL-2, IFNγ and TNFα) in these individuals and shown that IL-2, IFNγ and TNFα were significantly enhanced in active TB patients with DM compared to TB patients without DM [19]. We determined the relationship between the plasma levels of RAGE ligands in all TB participants with pro-inflammatory cytokines (Table 3). As shown, the systemic plasma levels of AGE showed a significant positive relationship with IL-2 and TNFα levels. In addition, sRAGE showed a significant positive relationship with IL-2, IFNγ and TNFα in all TB participants with and without DM at baseline, suggesting a significant association of these factors with cytokines.

**Table 3:** Relationship of RAGE ligands with pro-inflammatory cytokines

RAGE Ligands	IFNγ		TNFα		IL-2	
	r Value	***p*** Value	r Value	***p*** Value	r Value	***p*** Value
AGE	0.1661	0.1264	0.2403	**0.0259**	0.33	**0.0019**
sRAGE	0.2229	**0.0369**	0.2845	**0.0072**	0.287	**0.0067**
S100A12	0.1349	0.2101	0.1123	0.2974	0.135	0.2092
HMGB-1	−0.052	0.6336	0.0308	0.7757	−0.2448	**0.0215**

Statistical significant values are in bold

## Discussion

The growing prevalence of DM is a major global health challenge in its own right, and is further contributing to the global TB epidemic. Previous studies have described a detrimental interaction between DM and active TB [20–23]. DM also has a key adverse effect on TB treatment outcomes [15, 16, 22], including delayed sputum culture conversion, enhanced risk of treatment failure, and enhanced risk of TB relapse and mortality [24]. Published studies reveal that death attributable to TB is more prevalent in comorbid individuals after initiating anti-TB treatment, with a reported 5–7 fold increased risk overall and 17% mortality after 1 year compared to 7% in patients with TB alone [6, 25, 26]. Better understanding of TB-DM pathogenesis might lead to better therapies.

The RAGE receptor is expressed on diverse immune cell types including macrophages, neutrophils and lymphocytes, but the site of greatest expression is on the pulmonary epithelium [27]. As RAGE was first recognized as a receptor for AGEs, most of the clinical studies on RAGE are focused on the diabetic state [28]. Throughout an inflammatory response, RAGE–ligand interaction results in elevated expression of RAGE itself. This positive feedback loop results in continuous NF-kB activation, thereby altering a transient proinflammatory response into a chronic pathophysiological state [29]. A recently published study has stated that methylglyoxal, an AGE precursor, accumulates during *M. tuberculosis* infection and which in turn promotes macrophages apoptosis [30, 31]. During acute bacterial infections the impact of RAGE signalling in a non-diabetic state has been previously reported. RAGE deficiency has been shown to diminish the inflammatory response to both LPS stimulation and *E. coli* pneumonia in mice and drastically improve survival during bacterial sepsis [32, 33]. In addition, previously published studies reported the role of RAGE in pulmonary infections, which may correlate with TB since it is predominantly a pulmonary disease. Studies from murine models have also reported that RAGE is expressed in healthy lungs and are elevated after infection with *M.tb* and plays an vital role in chronic inflammation during tuberculosis [34]. Published studies from the murine models have shown that enhanced mortality was seen during tuberculosis in RAGE knock out mice, implying a protective role for RAGE ligands during murine tuberculosis [34]. With respect to clinical aspects, studies were reported that soluble RAGEs may exhibit ability for the identification of patients who are prone to the complications of diabetes and chronic hyperglycemia [35]. However, the role of RAGE or accumulation of AGE ligands in the context of TB-DM has been studied only in animal models but not in people [31]. Our data reveals that TB-DM and DM individuals displayed significantly increased circulating levels of AGE and sRAGE in comparison to TB and HC. In addition, before, during and after ATT, AGE and sRAGE levels remain persistently higher in TB-DM compared to TB. Our findings also exposed a unique relationship of AGE and sRAGE levels with the severity of TB disease (as estimated by the bilateral and cavitary disease) and with measured bacterial burden. Of further interest was our current results suggesting that AGE and sRAGE levels correlated positively with HbA1c, showing a relationship with poorly uncontrolled glycemia. Since diabetes has a significant impact on the expression of RAGE and its ligands, the role of RAGE signalling in infection may differ under diabetic conditions. In general, published data involve RAGE in the promotion of damaging inflammation, a process that is well accepted in diabetes, and therefore can also be rationally incriminated in the comorbidity of diabetes and infectious disease.

S100A12 is a member of the S100 low molecular weight family of calcium-binding proteins [36]. Human S100A12 is largely expressed and discharged by activated neutrophils [37]. Recent studies highlighted the involvement of S100 proteins with neutrophil related inflammation and their role as prospective surrogate markers to measure lung inflammation and disease severity in TB disease [38]. The S100A12 is the best accepted target protein of RAGE, and in this regard, S100A12 is known to elicit a pro-inflammatory immune response by binding to RAGE and activating transcription factors such as NF-kB [12, 37, 39, 40]. However, moderately high concentrations of these proteins are essential for RAGE activation, and they largely are engaged in chronic inflammation [41]. Published studies have reported that elevated serum levels of S100A12 are correlated with TB disease and that serum levels of S100A12 are also reported as good predictors of alveolar lung infiltration as assessed by chest X-ray [42]. In accordance, the results of our project demonstrate that TB-DM and DM individuals displayed significantly elevated circulating levels of S100A12 in comparison to TB and HC. In addition, before and during 2nd month ATT, S100A12 levels remained increased in TB-DM in comparison to TB. Our current findings also show an association of S100A12 levels with the severity of TB disease (as estimated by the bilateral and cavitary disease). Our results also warrant the conclusion that active inflammation in TB-DM results in increased S100A12 concentrations.

During the time of tissue injury, inflammation and infection, HMGB1, a DNA binding protein will be released from the nucleus and activates inflammatory and immune responses through binding to a group of receptors including RAGE and members of the TLR family [43, 44]. In addition, it is also reported that HMGB1 is released by macrophages, activated monocytes, platelets and neutrophils and in turn enables smooth muscle chemotaxis and proinflammatory responses of endothelial cells [45, 46]. Published studies have described that serum HMGB-1 circulating levels were increased in active TB patients compared to other lung disease or healthy controls, whereas in the same study no statistical differences were seen between the active TB and latent TB patients [47, 48]. In contrast to published studies, our results reveal that there are significantly diminished levels of HMGB-1 seen in TB-DM and TB in comparison to only DM individuals but no differences were seen between active TB disease and HC. In addition, our results also displayed no significant correlation of HMGB-1 with disease severity, bacterial burden and glycaemic status, indicating the lack of association between HMGB-1 with pathogenesis in TB-DM.

We have previously published that there was a bimodal representation of baseline HbA1c between KDM and NDM individuals in our study cohort, with significantly increased baseline HbA1c in the KDM group [16, 49]. In addition, we recently reported that matrix metalloproteinases (MMPs), which are important mediators of TB pathology were also significantly increased in KDM individuals compared to NDM [50]. Our current findings add to this clear heterogeneity in the presentation of TB-DM comorbidity. The findings from the current project report that systemic RAGE ligands, AGE and sRAGE were significantly enhanced in KDM in comparison to NDM groups, reflecting the enhanced diabetic severity in KDM individuals. Metformin is an oral antidiabetic drug that reduces hepatic gluconeogenesis and increases glucose uptake in skeletal muscle through its effect on the mitochondrial respiratory chain complex 1 and activation of the 5′-adenosine monophosphate-activated protein kinase (AMPK) [51]. A pioneering study reported that metformin may hinder the growth of *M.tb* and reduce lung inflammation in vivo by an AMPK-dependent mechanism [52]. Our group also recently reported that KDM participants on metformin treatment revealed a significantly diminished levels of MMPs [50]. Our existing results afford further evidence of a host-directed therapeutic effect for metformin based on diminished circulating levels of AGE and sRAGE.

The expression levels of RAGE, sRAGE, and several RAGE ligands including S100A12 and HMGB1 are known to be elevated in DM and to moderately correlate with poor glycemic control and the development of diabetic complications [53, 54]. Our findings demonstrate a correlation of plasma sRAGE and RAGE ligands with measures of TB disease severity, suggesting a possible underlying relationship. One of the limitations of our study is that we have not controlled for smoking, dietary intake, cardiovascular disease or chronic obstructive pulmonary disease, which might also affect the markers of inflammation. We have only measured CML and not the other AGEs.

## Conclusion

We identified that RAGE ligands were significantly enhanced in TB-DM and DM in comparison to TB and HC. We also demonstrated that bacterial burden in TB-DM was related to elevated circulating levels of RAGE ligands. Longitudinal follow-up of RAGE ligands reveal the alterations of these markers over the course of anti-TB treatment and provide evidence of unresolved inflammation at treatment completion in the TB-DM group. Our findings also suggest that RAGE ligands can distinguish KDM from NDM and are modulated by metformin therapy.

## Supplementary information

**Additional file 1.** The plasma levels of RAGE ligands were measured in TB individuals with bilateral and unilateral disease.

**Additional file 2.** The plasma levels of RAGE ligands were measured in TB individuals with cavitary and non-cavitary disease.

## Data Availability

All data generated or analysed during this study are included in this published article.

## Abbreviations

AGE: Advanced glycation end products
ATT: Anti-TB treatment
DM: Diabetes mellitus
DOTS: Directly observed treatment, short course
GM: Geometric mean
HbA1c: Glycated hemoglobin
HC: Healthy controls
HMGB1: High mobility group box 1
KDM: Diabetic before incident TB
LTBI: Latent TB infection
*M.tb*: *Mycobacterium tuberculosis*
NDM: Newly diagnosed with DM
sRAGE: Soluble RAGE
TB: Pulmonary TB
TB-DM: Tuberculosis-diabetes comorbidity
WHO: World Health Organization
