# Supplementary material for: Systemic RAGE ligands are upregulated in tuberculosis individuals with diabetes co-morbidity and modulated by anti-tuberculosis treatment and metformin therapy
Source: BMC Infect Dis. 2019 Dec 9;19:1039. doi: 10.1186/s12879-019-4648-1 (PMC6902343; doi:10.1186/s12879-019-4648-1)
Supplement: Supplementary file 1 — Additional file 1. The plasma levels of RAGE ligands were measured in TB individuals with bilateral and unilateral disease. [file 12879_2019_4648_MOESM1_ESM.docx]

Table S1: The plasma levels of RAGE ligands were measured in TB individuals with bilateral and unilateral disease

| **GeoMean** | **Bilateral** | **Unilateral** | **pValue** |
| --- | --- | --- | --- |
| **AGE (pg/ml)** | 2.88 | 3.27 | p=0.3359 |
| **sRAGE (pg/ml)** | 309 | 390 | p=0.2132 |
| **S100A12 (pg/ml)** | 1332 | 1238 | p=0.8707 |
| **HMGB-1 (pg/ml)** | 35 | 33 | p=0.3473 |
