# Supplementary material for: Systemic RAGE ligands are upregulated in tuberculosis individuals with diabetes co-morbidity and modulated by anti-tuberculosis treatment and metformin therapy
Source: BMC Infect Dis. 2019 Dec 9;19:1039. doi: 10.1186/s12879-019-4648-1 (PMC6902343; doi:10.1186/s12879-019-4648-1)
Supplement: Supplementary file 2 — Additional file 2. The plasma levels of RAGE ligands were measured in TB individuals with cavitary and non-cavitary disease. [file 12879_2019_4648_MOESM2_ESM.docx]

Table S2: The plasma levels of RAGE ligands were measured in TB individuals with cavitary and non-cavitary disease

| **GeoMean** | **Cavity** | **No Cavity** | **pValue** |
| --- | --- | --- | --- |
| **AGE (pg/ml)** | 3.04 | 3.06 | p=0.7998 |
| **sRAGE (pg/ml)** | 335 | 350 | p=0.5125 |
| **S100A12 (pg/ml)** | 1146 | 1349 | p=0.5760 |
| **HMGB-1 (pg/ml)** | 34 | 36 | p=0.7171 |
